# Development and Validation of the Protocol for Administering Telehealth Home (PATH) Assessments

**DOI:** 10.5195/ijt.2023.6545

**Published:** 2023-05-11

**Authors:** Cicily McBride, Sara Story, Jana Cason

**Affiliations:** 1 Spalding University, Auerbach School of Occupational Therapy, Louisville, Kentucky, USA; 2 Valparaiso University, College of Nursing and Health Professions, Valparaiso, Indiana, USA

**Keywords:** Aging in place, Home safety assessment, Occupational therapy, PATH protocol, Remote assessment, Synchronous home assessment, Telehealth

## Abstract

Home safety assessments and subsequent modifications can prevent hospitalization, institutionalization, and death among older adults. Telehealth has been shown to be an effective means to administer home safety assessments. However, a structured approach to synchronous telehealth home assessments has not been examined. This study involved development and validation of a structured telehealth home assessment protocol. The Protocol for Administering Telehealth Home (PATH) assessment was developed based on literature review, clinical experience with in-person and telehealth home assessments, and interviews with content experts. Six older adults participated in the validation phase of the protocol using a quasi-experimental, mixed-methods, one-group research design. The validation process resulted in modifications to the protocol to enhance its utility in practice. The PATH protocol provides a valid, structured approach to conducting synchronous telehealth home assessments and can be used by occupational therapists to administer home assessments for older adults desiring to age in place.

Rising health care costs coupled with health care provider shortages present a significant challenge to care for the health-related needs of the aging ‘Baby Boomer’ generation, people born between 1946 and 1964 (Haddad et al., 2022). Medicare and Medicaid are increasingly strained to fund this generation's expanding health care needs (Cubanski & Neuman, 2022; Werner & Konetzka, 2022). According to Kim et al. (2017), aging in place is “the ability to safely and comfortably maintain an independent and high quality of life in one's own home and community” (p. 25). Assisting this generation to age in place safely within their homes will decrease costs associated with hospitalizations and institutionalization.

Occupational therapists are experts in home safety assessments (HSAs). Occupational therapists are trained to identify safety concerns within the home and provide recommendations to enhance home safety. These modifications may include additional lighting, elimination of fall hazards (e.g., small decorative rugs, electrical cords), and installation of grab bars to prevent injuries and increase independence within the home. HSAs provided by occupational therapists, and subsequent modifications, can prevent hospitalization, institutionalization, and death (Stevens & Lee, 2018), decrease financial strain on individuals and health insurance providers (e.g., the Center for Medicare and Medicaid Services) (Werner & Konetzka, 2022), improve quality of life for older adults (Castillo et al., 2020), and reduce hours of formal and informal caregiver assistance (Carnemolla & Bridge, 2019).

Limited access to occupational therapists with expertise in HSA, travel times and associated expenses due to geographic distance between clients and therapists, as well as limited reimbursement results in few opportunities for older adults to receive HSAs (Renda & Lape, 2018). Synchronous telehealth (i.e., videoconferencing) is a solution to increase access to HSAs for older adults who desire to age in place safely within their homes.

Strong evidence supports home safety assessments provided by occupational therapists to increase safety and independence for older adults (Lau et al., 2018; Stark et al., 2017). There is also substantial evidence to support telehealth as a service delivery model for occupational therapy services, including home assessments (AOTA, 2018; Breeden, 2016; Dahl-Popolizio et al., 2020; Harkey et al., 2020; Nobakht et al., 2017).

In a study by Breeden (2016), clients were guided to take photographs of areas in their home that they perceived had safety challenges. These photographs were used to guide discussions on home safety, aging in place, and home modifications. The study employed the SAFER-HOME v.3 as an outcome measure with some improvement in home safety observed for five of six participants.

Gately et al. (2020b) conducted research focused on the feasibility of telehealth administered HSAs. Gately et al (2020a) also explored satisfaction of caregivers who assisted with the administration of synchronous telehealth HSAs for older veterans with dementia. The caregivers reported positive experiences with telehealth. However, caregivers reported that technical issues and the presence of the veteran in the home during the HSA had a negative effect on the overall experience. The researchers confirmed the feasibility of using telehealth to administer HSAs and concluded that having technical support available is important when using this service delivery model. Similarly, Read et al. (2020) found high client satisfaction with telehealth home assessments. Results indicate that telehealth home assessments are feasible and improve safety and perception of occupational performance (Breeden, 2016; Gately, 2020a; Gately 2020b; Read, 2020; Renda & Lape, 2018).

Research, though limited, has also explored telehealth HSA protocol development. Romero et al. (2018) developed and validated a remote home safety protocol for administering HSAs asynchronously. The protocol incorporated the Westmead Home Safety Assessment (WeHSA).

The aim of this study was to develop and validate a telehealth HSA protocol for synchronous telehealth administration of HSAs. The Protocol for Administering Telehealth Home (PATH) assessments provides a valid, structured approach to conducting synchronous telehealth home assessments.

## Methods

### Phase 1: PATH Protocol Development

Development of a synchronous home safety telehealth assessment protocol began with an exhaustive literature review related to HSAs, home modifications, telehealth, and HSAs conducted through telehealth. Content experts with extensive experience in telehealth home assessments were contacted for interviews. Information shared by these experts informed the protocol development process. In-person and virtual HSAs using the PATH protocol were completed by the first author to examine the protocol's utility in practice. Additionally, feedback from content experts and participants who received telehealth home assessments were used to further enhance the PATH protocol.

### Phase 2: PATH Protocol Validation

#### Study Design

Validation of the protocol was completed using a quasi-experimental, mixed methods, one-group research design. Data collected during the research study included participant demographics (i.e., age, sex, marital status), number of individuals in the home, home ownership, zip code, service delivery model (i.e., telehealth or in-person home assessment), type of device used during the assessment, inclusion of a care partner, technical issues (if any), duration of the home assessment, field notes related to implementation of the HSA, and feedback from home assessment participants via a survey. The research team consisted of an occupational therapy student completing a capstone project (CM), an occupational therapy (OT) capstone mentor with expertise in home assessments (SS), and a faculty supervisor with expertise in telehealth (JC). The research study was approved by Spalding University's Research Ethics Committee.

#### Study Procedures

Following recruitment, an initial phone call was made to each participant to collect general demographic information, as well as to administer a checklist to determine the appropriateness of the client for telehealth home assessment. It was determined that two of the six participants were not appropriate for telehealth. The primary reason for this determination was a lack of motivation by the individuals to complete a telehealth HSA. In addition, one participant did not have an email address and was concerned about downloading the videoconferencing software onto her smartphone. A second participant preferred an in-person home assessment but agreed to discuss the results of the HSA through telehealth (videoconferencing). Participants who were appropriate for telehealth HSAs were emailed an informed consent form to complete and return through email. Those who participated in an in-person HSA provided written consent.

Of the four participants who completed the telehealth HSA, only one agreed to have a practice session using the videoconferencing software prior to the session. All participants received a phone call reminder of their HSA 24-48 hours prior to the session. Zoom for Healthcare (Zoom, 2022), a HIPAA compliant videoconferencing software, was used for each telehealth HSA. Exit surveys were sent to the four telehealth HSA participants through QuestionPro.

The HSA consisted of two components. The first was an interview to create an occupational profile. The occupational profile focused on the client's concerns and priorities related to completing everyday tasks and participation in desired activities (AOTA, 2020). The occupational profile was an important component of the HSA, as it provided a holistic view of the client (e.g., likes, dislikes, leisure activities, and community involvements). This holistic viewpoint enables occupational therapy OT practitioners to find tailored solutions to clients' environmental concerns that hinder participation in meaningful activities.

The second component of the HSA consisted of evaluating the home environment. The Home for Life Design web application (https://homeforlifedesigndc.com/) was incorporated to provide a structured approach to synchronous telehealth HSA. Descriptive notes were recorded by the researcher. Photos of the home (e.g., rooms within the home and identified safety hazards) were obtained. For in-person HSAs, photos were taken with the researcher's iPhone 13 max. For telehealth HSAs, images were captured from the screen (i.e., screenshots) using a digital tool on the computer. Photos were used for reference when making recommendations for home modifications and were included in the Home for Life® report. Upon completion of the HSA, the Home for Life® report was electronically sent to the participants. The participants were instructed to review the Home for Life® report prior to completing the exit survey. The exit-survey was designed to obtain feedback from participants related to their experience with the telehealth HSA to inform further refinements of the PATH protocol.

#### Data Collection and Analysis

Data collected included feedback from clinical experts during the protocol development phase, demographic data from HSA participants, field notes, and survey data from participants obtained during the validation phase. Data was organized using Microsoft Excel. Additionally, photos and descriptive notes for each HSA were compiled in a Home for Life® report for each participant.

#### Participants

Convenience sampling was used to recruit participants in the research study to evaluate the validity of the PATH protocol. Individuals were informed about the study and had an opportunity to talk with the researcher. Participants were recruited in October 2022 through a religious organization. Inclusion criteria consisted of participants being 18 years or older and having access to a device with videoconferencing capabilities (e.g., smartphone, electronic tablet). Ten individuals were initially recruited. Three individuals were not available during the research period and one individual declined participation due to a spouse's illness. Six total participants (two male, four female) agreed to participate in the validation of the PATH protocol and completed an informed consent. See Table 1 for information related to participants' demographics, devices, and duration of HSAs.

**Table 1:** Participants' Demographics, Devices, and Duration of HSAs

Participants	Age (years)	Gender	Device	Duration of HSA (minutes)
Participant 1	78	F	N/A (in-person)	356
Participant 2	88	F	N/A (in-person)	140
Participant 3	79	F	MacBook Air Laptop	79
Participant 4	92	M	iPhone	88
Participant 5	80	F	Samsung Tablet	90
Participant 6	78	M	iPhone	65

### Expert Review of PATH Protocol

Five OT practitioners with expertise in telehealth and home assessments reviewed and provided feedback on the original version of the protocol to enhance its usability in clinical practice. Based on their review, several changes were made to the PATH protocol. Though not an exhaustive list, Table 2 outlines feedback incorporated into the revised protocol.

**Table 2:** Modifications to the PATH Protocol Based on Expert Review

Edited protocol organization and flow.
Replaced the term ‘caregiver’ with ‘care partner.’
Clarified some definitions (e.g., smartphone).
Due to liability concerns, removed suggestion that therapists provide clients with a list of reputable contractors.
Modified list of three common measurements for clients to take in each room.
Added link to an advertisement-free YouTube video with measurement instructions.
Provided alternate option to send pre-measured pieces of ribbon to facilitate measurement.
Clarified role of care partner if client presents with sensory or mobility deficits.
Added information and links for Home for Life® web application.

## Results

### Phase 1: PATH Protocol Development

#### Checklist to Determine Appropriateness of Client for Telehealth Home Assessment (Part 1 of the PATH Protocol)

Not all individuals are appropriate for telehealth HSAs. Therefore, Part 1 of the PATH protocol is a checklist to determine if the client is appropriate for a telehealth HSA (Appendix A). Questions include the client and/or care partner's motivation to participate in a telehealth HSA, ability to operate necessary technology, and client safety as it relates to participation in the HSA. The occupational therapist should use clinical reasoning informed by the checklist to determine if a client is appropriate for a synchronous telehealth home assessment.

#### Home Assessment Using the Home for Life® Web Application (Part 2 of the PATH Protocol)

To create a structured synchronous telehealth HSA protocol with good reliability, the Home for Life® web application tool was embedded into the PATH protocol. The Home for Life mobile and software solutions have been shown to have high inter-rater reliability (Lindstrom et al., 2019). The Home for Life® web application allows users to add client information (e.g., demographics), as well as create a personalized profile containing each room in the home that the therapist evaluates during the HSA. To aid in standardization, each room contains a personal safety score perceived by the client and room scoring as perceived by the therapist. These scores are used during re-evaluation to identify safety changes that occur after home modifications, assistive technology use, and client education are implemented.

The Home for Life® web application also allows for the inclusion of up to six photos of each room, as well as common home modification recommendations, some containing photo examples. Additionally, the therapist can generate a report of the assessment to share with the client or members of the healthcare team. Part 2 of the PATH protocol integrates the Home for Life® web application (see Appendix B).

#### Supplemental Resources (Part 3 of the PATH Protocol)

There are many different components to completing safe and effective telehealth home assessments. Thus, it was deemed necessary to include supplemental resources to provide additional guidance for therapists using the PATH protocol. Appendix C provides supplemental resources that comprise Part 3 of the PATH protocol. Appendix C1 provides information related to obtaining informed consent and pertinent laws and legal statutes. Appendix C2 contains helpful telehealth home assessment considerations (e.g., reminder to obtain the client's emergency contact information, device and connectivity considerations, information on HIPAA-compliant videoconferencing software, and basic technology problem-solving and home measurement strategies). Appendix C3 provides clinical, legal, and regulatory considerations for practitioners. Appendix C4 provides client educational material for telehealth home assessment with links to tech guides for setting up Android and Apple devices, creating an email account, and installing videoconferencing software. Appendices C5-C11 include client educational materials related to preparing for the telehealth HSA, safety tips, technology troubleshooting tips, and what to expect during the telehealth HSA. The materials were created in a format that allows therapists to download and customize the materials. See Table 3 for a list of PATH Protocol supplemental resources provided in Appendix C.

**Table 3:** PATH Protocol Supplemental Resources

Appendix C1	Informed Consent and Pertinent Statutes
Appendix C2	Helpful Telehealth Home Assessment Considerations
Appendix C3	Practitioner Considerations for Conducting Telehealth Home Assessments
Appendix C4	Telehealth Preparedness Checklist - 48 hours before appointment
Appendix C5	Telehealth Preparedness Checklist - 1 hour before appointment
Appendix C6	Telehealth Preparedness Checklist for Therapists
Appendix C7	Safety Tips for Telehealth Sessions
Appendix C8	Tech Troubleshooting Tips
Appendix C9	Important Videoconferencing Controls Infographic
Appendix C10	What to Expect from your Telehealth Home Safety Assessment
Appendix C11	Common Household Measurements

### Phase 2: PATH Protocol Validation

#### HSA Participant Feedback

Four HSAs were completed through telehealth and two HSAs were completed in-person, as per participant preference. Feedback from the telehealth HSA participants was positive. Three of the four participants commented that they were unsure how the HSA could be completed through telehealth but later were pleasantly surprised with the ease of the telehealth HSA. One participant commented that she felt more comfortable having a telehealth session with a doctor or other medical professional after participating in the telehealth HSA. Exit survey data from the four telehealth HSA participants revealed that they agreed the instructions provided before the home assessment helped them feel prepared and were easy to understand. All the participants agreed that the telehealth HSA increased their safety in their home and that they would recommend a telehealth HSA to a friend or family member.

On average, the in-person HSAs took 248 minutes (4 hours, 8 minutes) to complete compared to the telehealth HSAs which took 89 minutes (1 hour, 29 minutes). These times reflect the actual time conducting the HSAs and do not include additional documentation time completing the HSA report. The time for the in-person HSAs includes travel time to the participants' homes. The significant difference in time to complete telehealth HSAs versus in-person HSAs, including elimination of travel time, has clinical and economic implications.

#### PATH Protocol Modifications

Implementation of the PATH protocol for in-person and telehealth HSAs led to modifications to the protocol. Two of the six participants were deemed not appropriate for telehealth. As discussed, this was partially due to a lack of motivation to participate in a telehealth HSA. Therefore, a question related to motivation of the client and/or care partner to complete the HSA through telehealth was added to the checklist in Part 1 of the protocol.

Due to various technical issues that arose during the telehealth HSAs (e.g., clients having trouble connecting to audio, turning camera on, turning camera from front-facing to rear-facing, and internet connectivity issues), a stand-by second device was added for conducting telehealth home assessments. A second device was used by the researchers in three of the four telehealth home assessments to overcome loss of internet at the researcher's location (i.e., switching to cellular data rather than internet) and twice to talk the participant through the process of connecting audio after the participant had already joined by videoconferencing. To proactively address potential technical issues, an infographic using familiar icons was created. The infographic provides instructions on how to turn on and position the camera and how to mute/unmute oneself (see Appendix C9).

Another addition to the protocol was instructions for therapists on how to take photos of images on the computer screen (i.e., screenshots). This can be accomplished using a digital tool, such as Microsoft's Snipping Tool. It is important for therapists to take photos of the client's environment. Photos enhance documentation and are a component of the Home for Life® web application. Photos may also provide an objective view for the client when discussing the Home for Life® report and when identifying and discussing areas of concern within the home. The PATH protocol empowers therapists with knowledge on how to capture the client's environment through screenshots when providing telehealth HSAs.

Finally, two additional resources, a checklist for therapists and a checklist for clients, were added to the PATH protocol. These checklists were designed to help clients and their care partners, and therapists prepare for the telehealth HSA.

## Discussion

The purpose of this research study was to validate the newly created Protocol for Administering Telehealth Home (PATH) assessments. The protocol was developed to provide a valid, structured approach to conducting synchronous telehealth home assessments. Development of the protocol was informed by a comprehensive literature review, interviews with content experts, and clinical experience conducting in-person and telehealth HSAs. Six HSAs were completed using the PATH protocol, four through telehealth and two in-person. With recommendations from content experts during the PATH protocol development phase and information obtained from the PATH protocol validation phase, modifications were made to enhance the clinical utility of the PATH protocol.

The HSAs administered through telehealth took on average 1 hour and 29 minutes compared to 4 hours and 8 minutes, including travel time, for the in-person HSAs. The significant difference in time to complete the HSAs can be attributed to travel time for in-person assessments, as well as a more direct and efficient client interview and home evaluation through telehealth.

Participants reported in exit surveys that supplemental resources (i.e., client handouts) were helpful. These resources enabled clients to anticipate what to expect during the telehealth HSA, including the approximate duration of the HSA. Based on the information provided, one participant set up chairs in various locations around the home to assist with camera/electronic device placement so the researcher could easily view the participants' movement and transfers within the home. This participant completed the telehealth HSA independently without a care partner to assist with camera placement. The inclusion of a care partner was discussed with all telehealth HSA participants. The two participants who lived alone felt that they did not require a care partner's assistance to participate in the telehealth HSA. Clinical judgement was used by the researcher to support this decision. To maintain participants' safety, the researcher monitored participants' fatigue throughout the telehealth HSA. Participants were asked at least once during the assessment if a break was needed. One of the four participants accepted the break after ascending stairs to the second story of the home. Two participants chose to leave their pet outside during the telehealth HSA as an added precaution to improve safety (i.e., prevent falling/tripping over pet).

## Limitations

According to Romero et al. (2018), there is no “established methodology for validating instructional protocols to assist health professionals” (p. 171). Therefore, the protocol was validated using expert review by five content experts, as well as feedback from six home assessment participants after clinical implementation of the PATH protocol. The results indicate the protocol is valid and provides a structured approach to conducting synchronous telehealth home assessments. The PATH protocol can be used by occupational therapists to administer home assessments to older adults desiring to age in place as safely and independently as possible. However, several factors exist that limit generalizability of the findings.

First, the average age of HSA participants in the validation phase was 82 years (including the two care partners who assisted with the device/camera during the assessment). Most participants were female (n= 4) from one geographical region in the Southeast United States. All participants had some familiarity with videoconferencing as a result of its use during the COVID-19 pandemic. All telehealth HSA participants had videoconferencing software on their devices and had basic knowledge of its use prior to the study. This may have influenced participants' comfort with the technology and telehealth HSA.

Another factor affecting generalizability relates to participants' geographic and socioeconomic status (SES). All participants lived in a large urban city or its surrounding area. Individuals living in remote areas of the country or with lower SES may have limited access to high-speed internet and telehealth technologies (e.g., mobile devices, computers, electronic tablets) and may have less knowledge and experience with videoconferencing software (Hoel et al., 2021).

## Implications for OT Practitioners and Future Research

Validation of the PATH protocol demonstrates the feasibility of telehealth administered HSAs and provides a structured approach to completing synchronous telehealth HSAs. Telehealth HSAs promote health, well-being, and quality of life among older adults and prevent unnecessary hospitalizations and institutionalization related to falls in the home. Individuals being discharged from the hospital or an inpatient rehabilitation center to home, or who desire to age in place as safely and independently as possible may benefit from a telehealth HSA.

Additional research to explore the clinical utility of the PATH protocol with various populations in different geographical areas is needed. Populations may include individuals with disabilities, older adults with chronic health conditions, individuals post orthopedic surgery, and adults living in low-income neighborhoods. Additionally, feedback from occupational therapists using the PATH protocol could be used to further enhance the protocol.

## Conclusion

The use of telehealth to administer HSAs increases access to underserved individuals and is a partial solution to rising health care costs, shortages of health care practitioners, and an aging ‘Baby Boomer’ generation. Evidence supports telehealth as a feasible service delivery model for home safety assessments. Administering HSAs through telehealth supports older adults who desire to live as safely and independently as possible within their own home. The PATH protocol provides a valid, structured approach to conducting synchronous telehealth home assessments.

## Appendix A PATH Protocol Part 1: Checklist to Determine Appropriateness of Client for Telehealth Home Assessment

**Table:**

Is the client or care partner motivated to complete the home assessment virtually and complete any prep work that is involved (e.g., downloading software)	Yes	No	Unsure
Does the client or care partner have access to a mobile device (e.g., electronic tablet such as an iPad, smartphone with a camera) for the telehealth home assessment?	Yes	No	Unsure
Does the client or care partner have access to high-speed internet, cellular data, or hotspot to enable videoconferencing during the telehealth home assessment?	Yes	No	Unsure
Is the client or care partner able to operate the technology (e.g., turn on device, sign into videoconferencing meeting, and maneuver the camera to maximize visualization)?	Yes	No	Unsure
Can the client or care partner problem-solve and follow directions during the telehealth home assessment?	Yes	No	Unsure
Can the client (and care partner) safely maneuver around the home without fear of falling to complete the telehealth home assessment?	Yes	No	Unsure
Is an interpreter needed for the telehealth home assessment?	Yes	No	Unsure

*Note*. Using clinical judgment is crucial to determining the appropriateness of a client for a telehealth home assessment. The client may benefit from education or additional support (e.g., care partner) to participate in a synchronous telehealth home safety assessment safely and efficiently.

## Appendix B PATH Protocol Part 2: Home Assessment using the Home for Life Design® Web Application

The Home for Life Design (HFLD) web application (https://homeforlifedesigndc.com/) is a home safety assessment tool created by an occupational therapist. The application assists the therapist to evaluate and re-evaluate a client's home using checklists, photos, and safety ratings.

### Step 1: Complete Occupational Profile

Client information may include, but is not limited to, information about occupations, background, household, demographics, use of assistive technology, diagnoses/health conditions, problems with task performance, and fall and injury history. Inclusion of an additional assessment, such as the Canadian Occupational Performance Measure (COPM), is recommended.

### Step 2: Room Evaluation

Follow the instructions of the Home for Life® Web Application, for each room:

1. Score the client's safety and accessibility ratings.
2. Provide recommendations.
3. Describe the room with photos and notes.

### Step 3: Generate the Evaluation Summary Report

Share the evaluation summary report with the client and care partner, physician, or other authorized individuals. This report can be shared online using the web report link or downloaded as a PDF.

### Step 4: Identify and Discuss Suggested Modifications and Approximate Costs

Included within the evaluation summary report are the suggested home modifications, as well as other pertinent information. Identify and discuss all home modification recommendations with the client. Prioritize recommendations based on client preferences, occupational needs, and financial constraints.

Working effectively with other professionals ensures home modifications are completed appropriately. Interprofessional collaboration involves the client, care partners, contractors, tradespeople (plumbers, electricians, etc.), handypersons, volunteers, medical equipment professionals, and others (Siebert et al., 2014). Having a list of recommended professionals available to clients can improve implementation of recommendations (Lockwood, 2020). Any professionals recommended should be properly authorized, licensed, and insured (e.g., liability, worker's compensation, building risk). If you are unable to recommend contractors due to liability concerns, a good point of reference for the client is their local National Association of Home Builders (NAHB) group. Ideally, a hired contractor would be a Certified Aging in Place Specialist (CAPS), Certified Environmental Access Consultant (CEAC), or hold an Executive Certificate in Home Modifications.

### Step 5: Post-Home Assessment Re-evaluation

The Home for Life® Web Application has been found to have strong inter-rater reliability and has the capability to assess and document accessibility and safety ratings post home safety assessment (Lindstrom et al., 2019). It is recommended that the follow-up assessment is completed after home modification recommendations have been implemented and the client has been trained on their use. Reference the Home for Life® User Guide for detailed instructions for how to complete this within the web-based application.

Additional Home for Life® Resources:

- Home for Life® User Guide
- Scoring Decision Guide

## Appendix C1 PATH Protocol Part 3: Supplemental Resources

### Informed Consent and Pertinent Statutes

#### Guidance on Informed Consent

- Obtain consent, demographic information, payment information, etc., prior to beginning the telehealth home assessment. The informed consent form can be sent to the client using email, accessed through a Google Form (qualifications must be met to be HIPAA compliant), accessed through a website, or another HIPAA-compliant method of sending and receiving written documents.
- Verify client and/or care partner identity at the beginning of the telehealth home assessment with valid picture ID (e.g., driver's license or other government issued ID).

#### Related Statutes

The information below is quoted from *Telehealth occupational therapy services,* Section 2(7). Ky Stat. § 201 (2018) https://apps.legislature.ky.gov/law/kar/titles/201/028/235/

Inform the client in writing about:

- The limitations of using technology in the provision of telehealth occupational therapy services;
- Potential risks to confidentiality of information, or inadvertent access of protected health information, due to the use of technology in the provision of telehealth occupational therapy services;
- Potential risks of technology disruption during telehealth occupational therapy services;
- When and how the credential holder [occupational therapist] will respond to routine electronic messages;
- In what circumstances the credential holder [occupational therapist] will use alternative communications for emergency purposes;
- Who else may have access to client communications with the occupational therapist;
- How communications can be directed to a specific credential holder [healthcare practitioner];
- How the credential holder [occupational therapist] stores electronic communications from the client; and
- How the credential holder [occupational therapist] may elect to discontinue the provision of services through telehealth.

## Appendix C2 Helpful Telehealth Home Assessment Considerations

### Client Home Address

The client's home address is essential for alerting emergency personnel to the home in case of an emergency. In addition, paperwork or technology can be mailed to the client using the home address.

### OT Practitioner Business Address

The OT practitioner's business address and license information (i.e., state, license number) should be available to the client, as a mechanism to contact the agency or OT practitioner in writing if there are any complaints.

### Phone number

Should a technical issue or an emergency arise, having a phone number to contact the client or care partner is important. The OT practitioner should also have the phone number(s) of emergency personnel that are local to the client. Additionally, the client or care partner should have an alternate way to contact the OT practitioner (e.g., phone, email) should a technical issue or other concern arise prior, during, or after the telehealth home assessment.

### Phone/Tablet Requirements

A mobile device (e.g., smartphone or electronic tablet with video conferencing abilities) should be used during the telehealth home assessment to allow the client or care partner to move about the home with the device. A desktop computer or laptop is not recommended for home assessments due to their lack of mobility and size.

### WIFI or Hot Spot Requirements

Videoconferencing may be supported using high speed internet, cellular data, or a mobile hotspot. If using Broadband internet, connection speed should be 50-100 megabits per second (Mbps) (Let's Talk Interactive, 2021).

- At the time of this writing, the USAC Affordable Connectivity Program (https://www.affordableconnectivity.gov/) may assist with paying for broadband service and internet connected devices for those that qualify and provides:
  - Up to $30/month for internet service
  - Up to $75/month for those on tribal lands
  - One-time discount of up to $100 for a laptop, tablet, desktop

### Email Address

Assessment information, instructions, and access to the telehealth home assessment may be shared using email. If the client does not have an email account, consider assisting the client to create one.

### Care partner assistance

A care partner may be someone who lives in the home or is a friend, neighbor, or other support person who can assist the client during the telehealth home assessment. Inclusion of a care partner to assist is recommend, particularly if the client has trouble with vision, hearing, communication difficulties, or mobility limitations.

### HIPAA-compliant videoconferencing platform

A HIPAA-compliant videoconferencing platform should be used for the telehealth home assessment. There are many HIPAA-compliant platforms available (e.g., Zoom for Healthcare, Doxy.me, Simple Practice). Most of these types of platforms are fee-based for the practitioner. A Business Associate Agreement (BAA) should be provided by the platform in compliance with HIPAA requirements.

Information provided to the client prior to the telehealth home assessment should include a link to download the telehealth videoconferencing platform software, if required. Clients and care partners should be encouraged to test the technology and software prior to the telehealth home assessment. Practitioners should be aware that software looks different on different devices. Practitioners should become familiar with the functionality of the software on various devices to assist clients with technical questions.

### Client Tech Guides

Tech guides provided by Telehealth Access for Seniors

Guide for installing Zoom on an Apple device (iPhone or iPad)

### Basic technology problem-solving strategies

According to Health and Human Services (2021), basic tech problem-solving strategies include the following suggestions:

- Restart the computer or device.
- Make sure the device is fully charged.
- Check that the internet connection is working and the signal strength (e.g., WiFi, cellular) is strong enough to work with the telehealth platform.
- “Dead zones” are areas in the home where the internet may not work as well. Common areas include the basement or outside of the home. Issues with audio and video may occur. Attempt to change your location within the home to see if the problem resolves itself.
- Close all other applications.
- Use the most up-to-date version of Google, Chrome, Mozilla Firefox, or Safari (if the telehealth platform is web-based).
- If you are unable to connect to the telehealth platform, try connecting with a different browser and/or device.
- Contact a trusted IT professional that can assist with the issue.
- See Appendix C8 for a detailed infographic to share with clients and/or care partner.

### Taking Photos During a Telehealth Session

Photos are an important component of both in-person and virtual home safety assessments. Photos provide a reference for the therapist when documenting and can be uploaded into the HFL D application for inclusion in the client report.

**How to take a photo/screenshot on a Windows computer:**

- Hit the ‘PrtScn’ key simultaneously with the windows logo key.
- Photos will be saved in the ‘screenshots’ folder within the ‘pictures’ folder.
- Click here for a video with more information.

**How to take a photo/screenshot on a Mac laptop or desktop computer:**

- Hit the ‘shift’ key, ‘command’ key, and ‘3’ key simultaneously.
- Photos save to the desktop by default.
- Click here for a video with more information.

**How to take a photo/screenshot on an Android phone or tablet:**

- Press ‘power’ button and ‘volume down’ button simultaneously.
- Photos save to the photo gallery by default.
- Click here for a video with more information.

**How to take a photo/screenshot on an iPhone or iPad:**

- Press ‘power’ button and ‘volume up’ button simultaneously.
- Photos save to the photo gallery by default.
- Click here for a video with more information.

**Taking measurements**

Taking measurements is a standard practice of in-person home safety assessments. Areas that are typically measured include doorways, thresholds, the distance from the floor to an ideal placement for a grab bar, etc.

Determining if the client has a tape measure and knows how to use it is crucial for obtaining accurate measurements. You can refer the client to educational videos to educate them on the proper use of using a tape measure. Along with other instructional materials, you can also send a list of measurements for the client or care person to send to you before/after the appointment to guide recommendations (see Appendix C11).

Allowing the participants to assist with the home safety assessment process through measurement encourages buy-in and overall participation. If this is not feasible and your client does not own a tape measure, your options include:

- Using a dollar bill (6.14 × 2.61 inches)
- Using a standard piece of paper (8.5 × 11 inches)
- Mailing the client a tape measure or a pre-cut and pre-measured piece of ribbon
- Instructing the client to borrow a tape measure.

## Appendix C3 Practitioner Considerations for Conducting Telehealth Home Assessments

### Clinical Considerations

Practitioners should:

✓ Be in a private, quiet space for the telehealth home assessment.
✓ Test audio and video prior to connecting with the client.
✓ Have another device (smartphone, tablet, laptop) available in case you encounter technical problems.
✓ Confirm the identity of the client using picture identification.
✓ Confirm the client/care partner has your alternate contact information.
✓ Obtain the client's verbal consent (and written consent if required by state law).
✓ Discuss purpose and review plan for the telehealth home assessment.
✓ Determine who is present during the telehealth home assessment (e.g., client, care partner, other individuals).
✓ Address any client questions or technical concerns.
✓ Be cognizant of client and/or care partner fatigue (offer seated breaks, schedule additional sessions, encourage communication about fatigue).
✓ Review ‘Safety Tips for Telehealth Sessions’ with the client at the beginning of the telehealth home assessment (see Appendix C7 for a detailed infographic to share with clients and/or care partners).
✓ Use your therapeutic use of self to facilitate meaningful conversations during the telehealth home assessment. Difficult conversations may occur related to planning for the client's future home and health needs.

### Legal and Regulatory Considerations

The **OT Licensure Compact** is an interstate professional licensing compact for occupational therapy. It facilitates the interstate practice of occupational therapy by allowing occupational therapists and occupational therapy assistants to practice OT through telehealth across state lines in those states that have joined with the licensure compact (AOTA, n.d.; Occupational Therapy Licensure Compact, n.d.). When engaged in interstate OT practice, be cognizant of your client's time zone compared to yours when scheduling appointments. For more information on the OT Licensure Compact, visit: https://otcompact.org/about/

The **Fair Housing Act Amendments (1988)** is relevant for health professionals seeking home modifications for clients who rent in the private rental market. This act does not apply to those who rent from owner-occupied rental housing of four or fewer units or a single-family dwelling where the owner owns three or fewer units. The Fair Housing Act ensures tenants can make reasonable home modifications to their rental property to accommodate a disability, however the landlord is not required to pay for the modifications. The landlord is allowed to request the tenant to restore the space to move-in condition (i.e., remove modifications). For more information on the Fair Housing Act Amendments, visit: https://www.congress.gov/bill/100th-congress/house-bill/1158

The **Americans with Disabilities Act (1990)** is a civil law and does not apply directly to private dwellings. However, some funding sources require ADA compliance even in private dwellings. For more information on the Americans with Disabilities Act visit: https://adata.org/.

## Appendix C4 Telehealth Preparedness Checklist – 48 Hours Before Appointment^1^

**Table:**

**Telehealth Preparedness Checklist 48 hours (or more) before session**		
**Yes**	**No**	
		I have read the instructions provided to me by the therapist.
		I have identified and located a smartphone or electronic tablet with video conferencing capabilities. (A desktop computer is not appropriate).
		I have checked to make sure the device works and is fully charged.
		I have downloaded and tested the video conferencing software.
		The video is working.
		The audio is working.
		If your video and audio are NOT working, please reference the ‘Trouble Shooting Tips’ resource or contact your therapist directly.
		I have identified a care person (caregiver, family member, friend, neighbor) to help me during the session.
		If yes, I have contacted and confirmed the care person is available and willing to help me on the correct day and time of my session.
		I have a tape measure.
		If you DO NOT have a tape measure, locate a dollar bill, standard piece of paper, or contact your therapist.
		I have identified areas in my home where I have issues with connectivity on my mobile device (e.g., smartphone, electronic tablet).

## Appendix C5 Telehealth Preparedness Checklist – 1-Hour Before Appointment^2^

**Table:**

**Telehealth Preparedness Checklist 1-Hour Before Appointment** Check boxes as you complete items.	
	I have reviewed the safety tips sheet.
	I have gathered the necessary items (measuring tape, etc.).
	I have turned on my device and confirmed the battery is charged.
	I have logged into the video conferencing software 5 minutes before start of session.
	I have tested the audio and video to make sure they are both working.
	Once you have checked all the boxes, you are now ready to participate in the telehealth session.	

## Appendix C6 Telehealth Preparedness Checklist for Therapists

**Table:**

**Telehealth Preparedness Checklist for Therapists** Check boxes as you complete items	
	I have a primary device and a secondary device charged and ready for the telehealth session.
	I have headphones readily available if necessary.
	I have reviewed how to take digital photos (i.e., screenshots) during the telehealth session.
	I have received informed consent from the client and/or care partner.
	I have confirmed the identities of the client and/or care partner.
	I have reviewed the safety tips for the telehealth session (Appendix C7) with the client and/or care partner.

## Appendix C10 What to Expect from Your Telehealth Home Safety Assessment

- The goal of a home safety assessment is to identify areas within your home that could be improved to allow you to remain in your home as safely and independently as possible.
- The home safety assessment is expected to take approximately [insert anticipated timeframe].
- The home safety assessment may require multiple sessions.
- This session may be **performance based**. This means that you, as the client, will be required to perform transfers and simulate daily activities, such as getting in/out of bed and the shower or on/off the commode (fully clothed), in addition to showing the therapist around your home.
- The therapist will need an unobstructed view of you and your home. Using a phone stand or having a care person hold the device is recommended to ensure the therapist has an unobstructed view of your full body. You may also plan and identify areas where you might place your device during the session, such as on the bathroom sink or on a strategically placed chair.
- Be prepared to have difficult conversations with your therapist. Discussions around aging, worsening health conditions, and the future in general can be difficult. However, these conversations are important.
- Taking measurements is a standard practice of home safety assessments.
- If you have a measuring tape and are unsure how to use it, please watch this video (https://youtu.be/TWH8HVqkHP4)
- If you do not own a measuring tape and your therapist requested measurements, you can:
  - Purchase one at a local store or through an online retailer.
  - Use paper money or a standard piece of paper.
  - Request one be mailed to you.
- Session etiquette:
  - Please do not eat, take calls, or have unnecessary conversations with others in the household during your home safety assessment.
  - Turn off televisions and radios to limit distractions and unnecessary noise.

## Appendix C11 Common Household Measurements

Please review this list of common areas in your home. Take and write down the measurements that apply to your home. If you are unsure how to use a tape measure, please watch this instructional YouTube video (https://www.youtube.com/watch?v=TWH8HVqkHP4) or ask a care person for help. Your therapist may have additional instructions for you.

**Table:**

Area	Measurement
1. Height of toilet with seat down (all bathrooms)	
2. Width of main hallways	
3. Door jamb of bathroom (from inside of open door to door jamb)	
4. Door jamb of most used entrance to home	
5. From ground level to door threshold for all common entrances (i.e., front door, garage door)	
6. Other ____________________	
